# Automatic Recognition of Motor Skills in Triathlon: A Novel Tool for Measuring Movement Cadence and Cycling Tasks

**DOI:** 10.3390/jfmk9040269

**Published:** 2024-12-12

**Authors:** Stuart M. Chesher, Carlo Martinotti, Dale W. Chapman, Simon M. Rosalie, Paula C. Charlton, Kevin J. Netto

**Affiliations:** 1 Curtin School of Allied Health, Curtin University, Kent Street, Bentley, Perth, WA 6102, Australia; dale.chapman@curtin.edu.au (D.W.C.); kevin.netto@curtin.edu.au (K.J.N.); 2 Curtin Institute for Computation, Curtin University, Kent Street, Bentley, Perth, WA 6102, Australia; carlo.martinotti89@gmail.com; 3 SR Performance, Gairloch Drive, Frankston, Melbourne, VIC 3199, Australia; simon@srperformance.com; 4 Australian Institute of Sport, Leverrier Street, Bruce, Canberra, ACT 2617, Australia; paula.charlton@ausport.gov.au

**Keywords:** machine learning, peak detection, cycling task, cadence, motor performance, inertial measurement

## Abstract

**Background/Objectives**: The purpose of this research was to create a peak detection algorithm and machine learning model for use in triathlon. The algorithm and model aimed to automatically measure movement cadence in all three disciplines of a triathlon using data from a single inertial measurement unit and to recognise the occurrence and duration of cycling task changes. **Methods**: Six triathletes were recruited to participate in a triathlon while wearing a single trunk-mounted measurement unit and were filmed throughout. Following an initial analysis, a further six triathletes were recruited to collect additional cycling data to train the machine learning model to more effectively recognise cycling task changes. **Results**: The peak-counting algorithm successfully detected 98.7% of swimming strokes, with a root mean square error of 2.7 swimming strokes. It detected 97.8% of cycling pedal strokes with a root mean square error of 9.1 pedal strokes, and 99.4% of running strides with a root mean square error of 1.2 running strides. Additionally, the machine learning model was 94% (±5%) accurate at distinguishing between ‘in-saddle’ and ‘out-of-saddle’ riding, but it was unable to distinguish between ‘in-saddle’ riding and ‘coasting’ based on tri-axial acceleration and angular velocity. However, it displayed poor sensitivity to detect ‘out-of-saddle’ efforts in uncontrolled conditions which improved when conditions were further controlled. **Conclusions**: A custom peak detection algorithm and machine learning model are effective tools to automatically analyse triathlon performance.

## 1. Introduction

Triathlon is a sport where athletes swim, cycle, and run sequentially in a highly dynamic environment which requires them to have well developed continuous motor skills (i.e., swimming, cycling, and running) and discrete motor skills (i.e., cornering skills in cycling) to achieve elite performance [1]. To gain a detailed analysis of both continuous and discrete motor skills, three-dimensional motion analysis would be the most appropriate method. However, obtaining this level of detail during a race is logistically and feasibly complex to achieve with any certainty. Additionally, there is substantial inter- and intra-race variation, as races are conducted on courses with varying tidal conditions (swim), differing degrees of difficulty in cornering and elevation changes (cycle and run), and congestion caused by varying densities of triathletes [1]. Given these challenges, an alternative method for collecting and analysing data that describes motor skill performance in triathlon is required.

Measuring motor skill performance over time is important for identifying changes in triathletes’ motor skills so that intentional training strategies can be applied to improve performance. In triathlon, movement cadence refers to the number of propulsive movements performed within a specific time frame (usually one minute) [2,3,4]. In each discipline of triathlon, movement cadence has been identified as a parameter of swimming, cycling, and running motor skills which should be trained to achieve elite success [1]. Within an information processing paradigm, the ability to adjust the speed of movement to optimally fit the movement goal is a form of parametrisation [5]. Therefore, longitudinally measuring changes in movement cadence provides a measurement of a triathlete’s ability to parameterise the speed of swimming, cycling, and running motor skills, and as this improves, an improvement in motor skill performance can be inferred [6].

To better understand how movement cadence and other motor skills are performed differently in response to contextual racing factors (like contact with competitors and changes in elevation), it is essential to analyse them over small, contextually relevant timescales. For instance, a significant limitation of commercial sport watches is the inability to analyse movement cadence changes over short time epochs (e.g., 10 s or less). Furthermore, sport watches lack the positional accuracy required to meaningfully integrate stride frequency with global positioning data with high precision [7]. An investigation by Gløersen, Kocbach, and Gilgien [7] determined that a multi-sport wristwatch (Garmin Forerunner 920XT, Garmin International, Olathe, Kansas) had substantially larger error rates in detecting a horizontal position in space (2.54–3.28 m) than a trunk-worn inertial measurement unit (IMU) such as a Catapult Optimeye S5 (Catapult Australia, Melbourne) (0.34–0.51 m). These authors suggested that GPS antenna type, positioning (on the wrist vs. on the trunk) and sample rate (1 Hz vs. 10 + Hz) substantially compromise the accuracy of these sensors to collect GPS information [7].

Wearable IMUs provide a method of measuring the aspects of performance where video capture is not feasible. These wearable IMUs typically contain micro-electromechanical systems (MEMS), such as accelerometers, gyroscopes, and magnetometers, which measure along three axes as well as global position system (GPS) components [8]. By attaching these sensors to a body segment, information is gathered that can be used to infer the movements of the wearer. Wearable IMUs have been validated to analyse a variety of running and swimming performance metrics [9,10,11,12]. A recent investigation into the use of wearable IMUs in triathlon showed their validity for detecting swimming strokes, cycling pedal strokes, and running strides throughout a triathlon [13]. Although there have been several investigations of wearable IMU use in swimming and running, the automation of movement cadence measurement throughout an entire triathlon from a single wearable IMU is novel. Furthermore, it is important to verify that any performance information obtained by using automatic analysis methods is accurate and valid.

These investigators also explored the validity of the IMU to recognise cycling task changes, finding it to be a valid tool for recognising time spent ‘out of saddle riding’. However, there was a bias towards underestimating time spent ‘in saddle riding’ and overestimating time spent ‘coasting’. Although these biases were statistically significant (*p* < 0.001), the practical differences were small (in saddle riding: −0.45 s [−1.11 to 0.19 s]; coasting: 0.39 s [0.19 to 0.58 s]) [13]. However, the analysis in this investigation was performed visually, using a time- and labour-intensive method, without assistance from machine learning or automatic pattern recognition.

Human activity recognition by machine learning has been used in sports performance analysis as a fast and accurate method to describe and quantify important performance metrics [14,15,16,17,18]. To do this, wearable IMU data are analysed using machine learning algorithms to detect patterns that correspond to specific movement signatures. Analysing data in this way makes it possible to recognise complex patterns across multiple data streams and provide an analysis far quicker than manual analysis methods. Therefore, this investigation aims to advance the application of wearable IMUs in a sprint-distance triathlon (750 m swim, 20 km cycle, and 5 km run) by developing an automatic activity detection algorithm to detect swimming strokes, cycling pedal strokes and task changes, and running strides performed in a race.

## 2. Materials and Methods

### 2.1. Participants

Six triathletes (participants 1–6; three females and three males; mean age = 16.2 yrs. ± 2.7; competition level: one Tier 4: elite/international; four Tier 3: highly trained/national; and one Tier 2: trained/developmental [19]) were purposively sampled to participate in a mock triathlon on a course constructed for the research study. These participants were selected to form a heterogenous sample of triathletes with a variety of skill levels, ages, and statures. Sampling in this way will establish a deeper understanding of the ecological validity of the wearable IMU and activity recognition algorithm compared to a homogenous sample. Triathletes were eligible to participate if they were at least 12 years of age, could swim continuously for 200 m, cycle for 10 km, and run for 2 km, and were excluded if they had any injury preventing participation in training or competition. Written informed consent was provided by all participants over 18 years old, while parental consent and participant assent was obtained for those under 18 years old. Ethical approval for this research was granted by the institution’s human research ethics committee (HRE2022-0048).

### 2.2. Methodology

The methodology used in this study is the same as those used by Chesher, Rosalie, Chapman, Charlton, van Rens, and Netto [13] to investigate the validity of this measurement tool using manual analysis methods. To collect movement data, participants completed a triathlon on a specifically constructed racecourse. During the triathlon, each participant was filmed by a paired volunteer while wearing a trunk-mounted wearable IMU (Optimeye S5, Catapult Innovations, Melbourne, Australia) to collect movement data. Prior to the triathlon, participants set out their equipment (bike, equipment box, and running shoes) as they would in a race, then completed a ten-minute warm up consisting of low-intensity sport-specific activities and dynamic stretching. Participants were then briefed on the course, which consisted of one lap of a 400-metre swim course in a lake, a transition area, four laps of a five-kilometre elongated rectangular cycling course (20 km), and two laps of a 2.5 km run course along a footpath (5 km), finishing back at the transition area. Participants began the triathlon in a time-trial format, each beginning 30 s apart to prevent the visual obstruction of the video cameras.

To confirm the occurrence of swimming strokes, pedal strokes and task changes, and running strides, participants were filmed using stationary cameras (CasioEXZR-800, Exilim, Tokyo, Japan) attached to tripods, and mobile cameras (Hero Session 5, GoPro, San Mateo, CA, USA) attached to the handlebars of bikes ridden by the paired volunteers. Camera set-up and filming technique were briefly piloted during a group training session prior to data collection. Sensor data and video footage were time synchronised at the start of each triathlon segment by striking the sensor five times in view of the video camera to create distinct peaks in the forward accelerometer signal. The wearable IMU contained a GPS sensor, tri-axial accelerometer, and gyroscope, which measured 96.5 × 52 × 14 mm^3^, and weighed approximately 67 g and was positioned between the shoulder blades in a custom-made pouch within the triathlon suit. The GPS sampled at 10 Hz, while both the accelerometer and gyroscope sampled at 100 Hz along three axes with measurement ranges of ±16 g and 2000°/s, respectively. Following the triathlon, both video and wearable IMU data were analysed to count the swimming strokes, pedal strokes, and running strides, and to record the time stamp and the duration of cycling task changes.

To obtain a ground truth value for swimming strokes, pedal strokes, and running strides, the video footage was analysed (Avidemux 2.8; Mean, Gruntster and Fahr; Paris, France), and the movement cadence in each discipline was manually counted and recorded on a custom spreadsheet (Excel 2019, Microsoft Corporation, Redmond, WA, USA). Swimming strokes were counted when any part of the upper limb from fingertips to elbow entered the water, pedal strokes were counted when each of the participant’s feet revolved to the bottom of the pedal crank (i.e., there are two pedal strokes in one entire revolution), and running strides were counted at each heel strike.

Cycling task changes were identified by viewing the footage, and the start and end times of each task were recorded. ‘In-saddle’ riding was defined as pedalling while the gluteus maximus was in contact with the bike seat, ‘out-of-saddle’ riding was defined as pedalling without contact between the gluteus maximus and the seat, and ‘coasting’ was defined as riding without actively turning the pedals for more than one second, regardless of seat contact.

**Figure 1:**
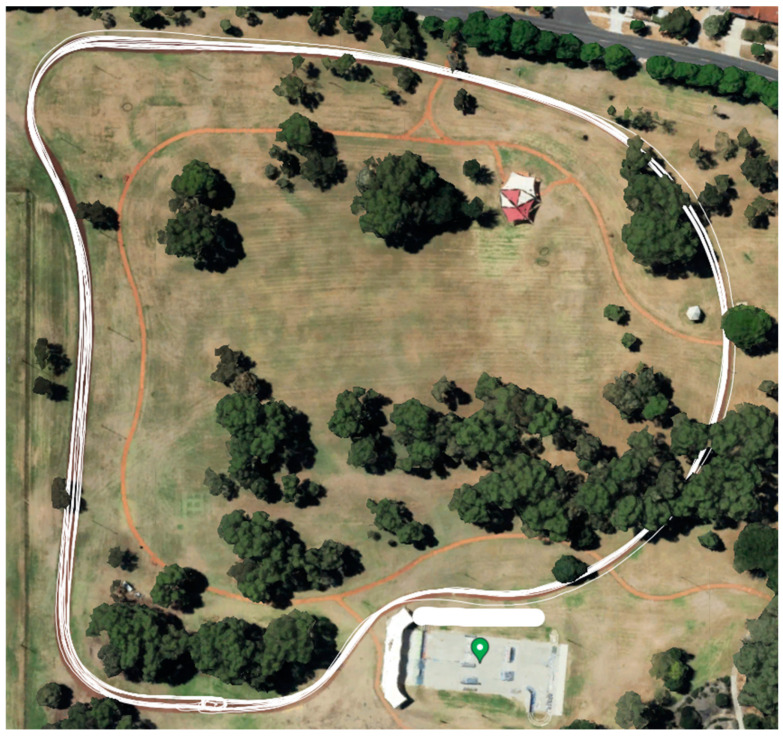
Map of course used for additional cycling data collection.

From the initial analysis, ‘in-saddle’ riding was substantially overrepresented (90–94% of the duration) compared to ‘out-of-saddle’ riding (1.5–7.8%) and ‘coasting’ (1.8–4.5%) throughout the triathlon. To create a valid machine learning algorithm that recognises each cycling task, additional controlled data with clearly demarcated and evenly represented cycling tasks was required. Thus, 217 min of additional cycling data were collected from six participants (participants 7–12; four female and two male triathletes; mean age = 20.33 yrs. ± 3.3; competition level: two Tier 4: elite/international level; two Tier 3: highly trained/national level and two Tier 2: trained/developmental level [19]) who cycled around a closed track, performing ‘in-saddle’ riding, ‘out-of-saddle’ riding, and ‘coasting’ in a specific sequence for equal 20 s durations. Durations of this length were chosen as it was suspected that the parameters of the short-time Fourier transform blurred the signal of short duration (2–4 s) cycling tasks. A 180° turn was included at the end of each lap to ensure an even distribution of turn directions. A different track was used for the second round of data collection (Figure 1) to allow for greater control of the conditions, improving the quality of the data for training the machine learning algorithm. Additionally, conducting the second round of data collection on a different track enhanced the model’s validity, generalisability, and robustness to noise [20].

### 2.3. Creating the Performance Analysis Tool

To automate the detection of swimming strokes, cycling pedal strokes, task changes, and running strides, both a ‘peak counting’ and a machine learning model were created. ‘Peak counting’ refers to counting the peaks and troughs in the accelerometer and gyroscope signal that correspond to swimming strokes, pedal strokes, and running strides. To begin, the accelerometer signals were filtered using a sixth-order bandpass Butterworth filter with lower and upper cutoff frequencies of 2 Hz and 3 Hz, respectively, for cycling and running. The Butterworth filter was chosen for its maximally flat passband response and gradual roll-off from the passband to the stopband, which allows it to effectively remove unwanted frequencies while preserving the desired ones [21]. For swimming, the same filter was used, but with lower and upper cutoff frequencies of 0.5 Hz and 1.4 Hz due to the slower movement cadence of swimming. Peaks and troughs of the filtered accelerometer signal were counted using SciPy (2022, version 1.9.2, Enthought, Austin, TX, USA). The minimum peak detection intervals for each movement were set at 0.5 s for swimming, 0.3 s for cycling, and 0.25 s for running.

Next, a machine learning model was built to classify cycling tasks. The accelerometer and gyroscope signals were combined and filtered using the same method as for peak counting. A short-time Fourier Transform (STFT) was then applied using SciPy to convert the time-domain signal into the frequency domain. Signal processing by STFT is commonly used to analyse distinct patterns in signal data [22]. The frequency content of accelerometer signals generated by physical activity can change over time, making time-frequency domain analysis more appropriate [23]. A window size of 250 samples (2.5 s) with no overlap was chosen, resulting in 126 frequency bins for each time step, which were used as additional features for model training. Data standardisation was then performed using the ‘standard scaler’ function in Scikit-learn (2023, version 1.2.2, Cournapeau, D.). Cycling task classification labels were aligned with the original time series, featuring 0.1 s steps, and with the resampled series from the STFT with 2.5 s steps. For each longer step, the most frequent label from the 250 shorter steps was assigned to the long step [23]. Finally, an XGBClassifier was trained using XGBoost (2023, version 1.7.5, Xu, B.) in Python (2023, version 3.12.0b3, Python Software Foundation, Wilmington, DE, USA). Since this work serves as a proof of concept rather than a fully optimised model, default parameters were used, and fine-tuning was reserved for future investigations. The resulting data were then exported to Excel (version 2305, Microsoft, Redmond, WA, USA) for analysis and visualised onto a map using the folium library in Python.

Descriptive statistics were calculated to show the average and standard deviation of the number of swimming strokes, pedal strokes, and running strides for each participant. To evaluate the accuracy of the peak-counting algorithm and the machine learning model, the percentage of correctly counted swimming strokes, cycling pedal strokes, running strides, and cycling task classifications were calculated [20]. To evaluate the error and provide practical interpretation, the root mean square error (RMSE) and relative error were calculated for the peak-counting algorithm. Subsequently, as cycling task is a categorical variable, the sensitivity and specificity was calculated for the machine learning algorithm to detect the correct labelling of data points as ‘out of saddle’ riding [20]. Three participants from the first round (participant one, two, and three) and three participants from the second round (participant eight, nine, and eleven) of data collection were randomly selected, and their data was used to train the cycling task recognition model using the ‘hold out’ method [24]. With this method, 80% of the data from these six participants were used for training and the remaining 20% were used for testing. The remaining six participants were entirely excluded from the model training process and used solely to test the generalisability of the model.

## 3. Results

The peak-counting algorithm successfully counted swimming strokes, pedal strokes, and running strides. The ground truth average number of swimming strokes, pedal strokes, and running strides per participant was 341 swimming strokes (±43), 2760 cycling pedal strokes (±171), and 2036 running strides (±568), respectively. During cycling, participant five’s wearable sensor came loose from the race suit, resulting in a corrupted cycling accelerometer signal. Consequently, these data were removed and reported as “N/A”. The percentage accuracy of the peak-counting algorithm is presented in Table 1.

**Figure 2:**
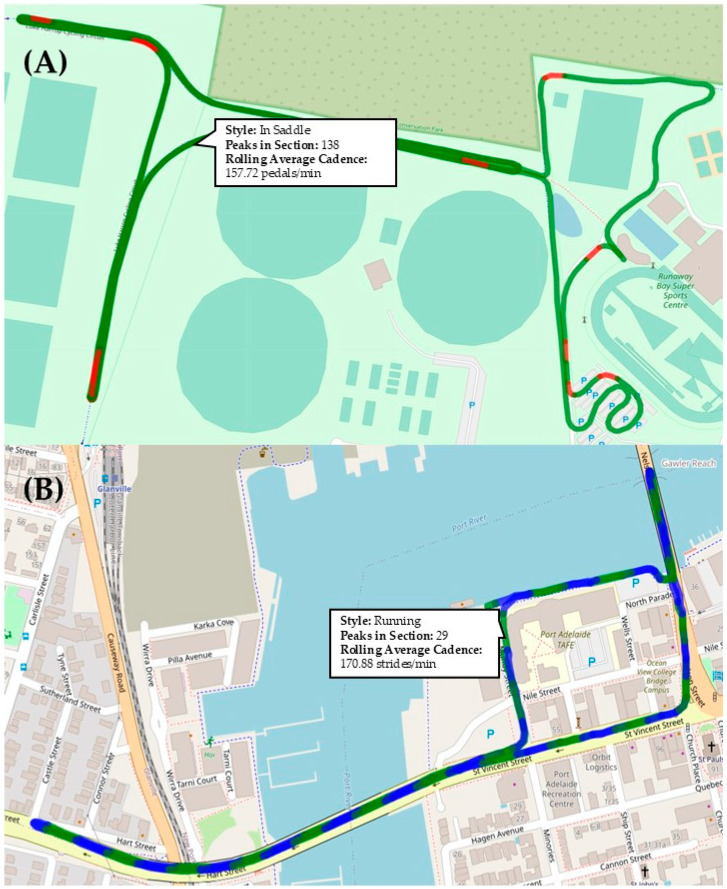
(**A**) Cycling task classification and (**B**) running performance map. Note: The red sections of the cycling performance map (**A**) show the location and duration of ‘out-of-saddle’ riding and the green sections show ‘in-saddle’ riding. On the running performance map (**B**), the green and blue sections indicate intervals of customisable length where the number of running strides during those intervals is calculated along with a rolling average of stride rate.

The average swimming cadence across all participants and races was 78.9 (±8.1) strokes/min with a relative error of 3.4%. The average cycling cadence across all participants and races was 157.5 (±6.6) pedal strokes/min with a relative error of 5.8%, and the average running cadence across all participants and races was 172 (±5.9) strides/min with a relative error of 0.7%. The average ground truth of cycling task changes for the original dataset was 21 (±5.6) instances of ‘in-saddle’ riding (16.27 min ± 13.8 s), 11 (±5.2) instances of ‘out-of-saddle’ riding (37.7 ± 21.0 s), and 11 (±2.6) instances of ‘coasting’ (28.1 ± 10.3 s). For the additional cycling data collection, the average ground truth was 67 (±18.3) instances of ‘in-saddle’ riding (12.6 ± 2.6 min; 34.9%), 42 (±8.6) instances of ‘out-of-saddle’ riding (11.5 ± 2.5 min; 31.9%), and 60 (±18.5) instances of ‘coasting’ (11.7 ± 2.8 min; 32.3%). The machine learning model was not accurate at distinguishing the sections of ‘coasting’ from ‘in-saddle-riding’. Therefore, only the accuracy for distinguishing between ‘in-saddle’ and ‘out-of-saddle’ riding has been reported (Table 2) and visualised on the cycling task classification map (Figure 2A).

Finally, the machine learning model was designed to plot the race performance from participants on a map with changes in cycling tasks and revolutions per minute information available for the analysis of cycling and a run course with a stride rate at customisable intervals available. Examples of the performance of the model are available in Figure 2.

## 4. Discussion

This study investigated the accuracy of a peak detection algorithm and a machine learning model to calculate movement cadence across the three disciplines of triathlon, and to classify cycling task changes during the cycling leg of the race. In swimming, comparing the RMSE with the sample standard deviation shows that the expected error range is ±0.33 standard deviations from the mean, giving an expected range of 74.3 to 79.7 strokes/min for an elite open-water swimmer with a medium cadence (77 strokes/min) [25]. This indicates a high degree of accuracy. In cycling, comparing the RMSE with the sample standard deviation shows that the expected error range is ±1.38 standard deviations from the mean. For an elite triathlete cycling at an average cadence (194 pedals/min or 97 RPM), the expected range is 185 to 203 pedals/min (or 92.5 to 101.5 RPM). Thus, the accuracy is lower than in swimming, but the error range remains practically useful. Finally, in running, comparing the RMSE with the sample standard deviation shows that the expected error range is ±0.2 standard deviations from the mean, giving an expected range of 180.8 to 183.2 strides/min for an elite triathlete with a cadence of 182 strides/min [26] which is considered highly accurate.

These findings contrast slightly with previous research which showed lower error rates when using the visual inspection of inertial sensor signals to measure triathlon movement cadence [13]. Chesher, Rosalie, Chapman, Charlton, van Rens, and Netto [13] found that swimming strokes, cycling pedal strokes, and running strides could be detected with very high accuracy (−0.2 swimming strokes, −0.5 cycling pedal strokes, and 0 running strides per minutes) as well as ‘out-of-saddle’ riding (0.08 s, respectively). While there has been some reduction in accuracy to automate movement cadence measurement, the ranges for error are still practically useful to use as a tool that enables the assessment of a triathlete’s ability to parameterise the speed of swimming, cycling, and running motor skills.

The machine learning model developed to detect transitions between cycling tasks successfully differentiated between ‘in-saddle’ and ‘out-of-saddle’ riding but was inaccurate at distinguishing between ‘in-saddle’ riding and ‘coasting’. While the algorithm exhibited high accuracy in identifying correctly labelled time points, the sensitivity and specificity analysis offers a more nuanced interpretation. Specificity across all participants was high, indicating that the model was effective at recognising ‘in-saddle’ riding. However, the low sensitivity revealed its poor performance in detecting ‘out-of-saddle’ riding. Notably, there is a distinct difference in specificity between the first (participants 1–6) and second (participants 7–12) rounds of data collection by 43.4%, contrasting with the algorithm’s measured accuracy. Two factors likely explain this discrepancy: (1) In the first round, ‘in-saddle’ riding was overrepresented (90–94% of riding time) compared to ‘out-of-saddle’ riding, meaning that the algorithm’s high specificity inflated its overall accuracy, misrepresenting its ability to detect ‘out-of-saddle’ efforts. (2) In the first round of data collection, ‘out of saddle’ riding efforts were short (~2–4 s) compared to the imposed duration of 20 s in the second round of data collection. As the short-time Fourier transform used a window size of 2.5 s, this blurred shorter ‘out-of-saddle’ efforts, reducing detection accuracy.

The automation of cycling task analysis using the machine learning model has contrasting accuracy compared to previous research [13]. However, as in earlier work, the model still failed to distinguish ‘coasting’ from ‘in-saddle’ riding, likely due to the small amplitude differences between the two tasks, compounded by the sensor’s torso placement, which attenuates reaction forces through the kinetic chain—a finding echoed in running studies [27]. To improve the accuracy of the model, a more suitable approach for differentiating these tasks may involve using a convolutional neural network (CNN) to analyse the signal’s shape or integrating a pedal crank power sensor. However, the current dataset was too small for CNN analysis, a limitation that should be addressed in future research.

Another way to improve cycling task recognition would be to alter the window size of the short-time Fourier transform. Selecting an appropriate window size is important to balancing the time and frequency resolution. When trying to analyse a signal with rapidly changing frequency, a shorter window size can more accurately track these changes, compared to averaging over a longer window. As the minimum cycling task length in this investigation was one second, a reduced window size may be more accurate to distinguish between rapid changes in cycling tasks [28].

Collecting objective data during triathlons has previously been challenging due to the logistical complexity of video capture and the lack of validated wearable IMU technologies. In some cases, performance analysts manually analyse multiple data streams obtained from multiple sensors attached to various body segments to gain insights. Therefore, this research fills a gap in triathlon performance analysis by quickly generating nuanced performance insights from a single IMU that measures global position more accurately than common alternatives [7]. A wearable IMU positioned on the trunk is unlikely to capture kinematic information about the lower body due to its distance from the relevant segments. However, the use of a lower-body attachment location for a wearable IMU has been investigated to measure the number of kicks performed while swimming [29]. This investigation found that a wearable IMU has high accuracy and sensitivity to measure the number of kicks performed during freestyle swimming.

At a basic level, this research offers a simple performance analysis tool that measures changes in movement cadence and cycling tasks over time. However, its application could be expanded to assess motor skill adaptability, a key factor in elite triathlon success [1]. Although swimming, cycling, and running are typically considered “closed” motor skills, their execution in a triathlon context transforms them into more “open” motor skills that require adaptability to varying contextual factors, such as racecourse features and environmental conditions [30]. Evaluating this adaptability necessitates measuring performance changes over timescales comparable to those of the contextual variations. Thus, measurements of movement cadence per minute, or average movement cadence per kilometre are inappropriate to evaluate motor skill adaptability.

Plotting changes in motor skill performance over time can identify changes in the quality, consistency, and stability of swimming, cycling, and running motor skills [6]. Furthermore, combining performance data and contextual information can show persistence of progress and reduced attention demand in motor skill performance [6]. For example, Bouillod and Grappe [31] identified that some cyclists alternate between ‘in-saddle’ and ‘out-of-saddle’ riding to maintain speed during a race despite the increased mechanical cost of ‘out-of-saddle’ riding. While this could be influenced by environmental features imposed by the race (hills, overtaking, corners), it may also reflect a cyclist’s difficulty in maintaining an efficient in-saddle riding motor pattern [1,31]. This could indicate that these cyclists have not learned the stability of the cycling motor pattern at that speed. Thus, measuring time spent in different cycling positions and linking it to racecourse features like elevation could assess motor skill stability.

This investigation addresses a gap in the literature by developing a practical measurement tool capable of recording movement cadence across all three triathlon disciplines using a single sensor, eliminating the need to merge data from multiple sources. The high accuracy and relative simplicity of this method of performance analysis makes it suitable to implement in a practical setting. The peak-counting algorithm and map visualisation provides coaches and sport scientists the ability to analyse each discipline in short time intervals and detect performance changes caused by environmental features like hills, corners, competitor congestion, and tidal conditions. This nuanced and detailed performance data can inform coaches’ training decisions, enabling incremental improvements that are especially valuable at the elite level.

Some limitations should be considered when interpreting this research. First, the machine learning model could not distinguish between efforts of ‘coasting’ and ‘in-saddle-riding’, limiting the strength of the inferences that can be made about cycling motor skills related to cornering or pedalling consistency [1]. Second, the peak-counting algorithm and cycling task recognition model cannot yet be generalised, as it was developed from a small subset of triathletes and a larger, more diverse dataset is required for generalisation.

Further research should aim to continue developing the cycling task recognition model to distinguish ‘coasting’ from ‘in-saddle’ cycling to deepen the analysis that can be performed. Zignoli and Biral [32] note that cyclists typically adopt one of two cornering strategies: maintaining a high velocity but also a large radius of curvature (and thus travel a greater distance) or take a shorter path with greater velocity loss. The latter strategy is commonly utilised, but requires a precisely timed late braking point (coasting) and an early high-power ‘out-of-saddle’ effort [32]. Measuring this could be possible by combining the peak-counting algorithm and the machine learning model to determine ‘coasting’ as ‘in-saddle’ riding with no pedalling. This proof of concept for trunk-mounted wearable sensors could be integrated with bike computers to combine GPS, power, and inertial data for a comprehensive time-motion analysis of cycling. Additionally, further research should investigate the average changes in the movement cadence of youth triathletes over time to inform training practises.

## 5. Conclusions

Developing a peak-counting algorithm to measure cadence at customisable intervals, along with a machine learning model to recognise cycling task changes, is an important step towards improving motor skill analysis and the practise design in triathlon. Automating this process also makes the analysis of multiple athletes feasible, given the time and resource constraints faced by many elite sporting organisations. Further refinement of the peak-counting algorithm to include additional performance metrics, and enhancement of the machine learning model to recognise ‘coasting’ efforts can deepen coaches’ and sports scientists’ understanding of their athletes’ performance without requiring additional analysis time.

## Figures and Tables

**Table 1:** Accuracy of peak detection algorithm in each discipline of triathlon.

	Swimming Strokes		Cycling Pedal Strokes		Running Strides	
	% Accuracy	RMSE (str/min)	% Accuracy	RMSE (str/min)	% Accuracy	RMSE (str/min)
P1	99.1%	2.0	98.0%	12.0	98.7%	1.0
P2	99.1%	2.9	98.6%	9.5	98.5%	2.6
P3	98.4%	1.3	98.2%	7.3	99.7%	1.5
P4	99.5%	5.4	96.1%	7.9	99.9%	0.7
P5	98.2%	3.7	N/A	N/A	99.9%	0.6
P6	98.2%	1.0	97.9%	8.7	99.8%	0.8
Average	98.7% (±0.5%)	2.7 (±1.5)	97.8% (±1.0%)	9.1 (±1.6)	99.4% (±0.6%)	1.2 (±0.7)

**Table 2:** Accuracy of machine learning model to recognise cycling tasks.

Participant #	Percentage Accuracy	Sensitivity	Specificity
P1	97.6%	42.2%	95.7%
P2	100%	32.4%	97.6%
P3	98.8%	22.5%	99.6%
P4	98.8%	10.5%	99.5%
P5	N/A	N/A	N/A
P6	91.5%	63.4%	98.5%
P7	94.7%	86.4%	93.5%
P8	97.6%	87.0%	93.9%
P9	91.7%	83.1%	92.6%
P10	91.5%	85.5%	91.9%
P11	83.3%	52.5%	96.9%
P12	88.1%	70.3%	98.3%
Average	94.0% (±5.0%)	57.8% (±26.4%)	96.2% (±2.7%)

## Data Availability

Data are available upon request by contacting the corresponding author (privacy and ethical).

## References

[1] Chesher S.M., Rosalie S.M., Netto K.J., Charlton P.C., van Rens F.E.C.A. (2022). A qualitative exploration of the motor skills required for elite triathlon performance. Psychol. Sport Exerc..

[2] Ribeiro J., Figueiredo P., Morais S., Alves F., Toussaint H., Vilas-Boas J.P., Fernandes R.J. (2017). Biomechanics, energetics and coordination during extreme swimming intensity: Effect of performance level. J. Sports Sci..

[3] Moore I.S. (2016). Is there an economical running tehnique? A review of modifiable biomechanical factors affecting running economy. Sports Med..

[4] Turpin N.A., Watier B. (2020). Cycling biomechanics and its relationship to performance. Appl. Sci..

[5] Schmidt R.A. (1975). A schema theory of discrete motor learning. Psychol. Rev..

[6] Magill R., Anderson D. (2014). Motor Learning and Control: Concepts and Applications.

[7] Gløersen Ø., Kocbach J., Gilgien M. (2018). Tracking perforance in endurance racing sports: Evaluation of the accuracy offered by three commercial GNSS receivers aimed at the sports market. Front. Physiol..

[8] Crang Z.L., Duthie G., Cole M.H., Weakley J., Hewitt A., Johnston R.D. (2021). The validity and reliability of wearable microtechnology for intermittent team sports: A systematic review. Sports Med..

[9] Mooney R., Corley G., Godfrey A., Quinlan L.R., ÓLaighin G. (2016). Inertial sensor technology for elite swimming performance analysis: A systematic review. Sensors.

[10] Ganzevles S., Vullings R., Beek P.J., Daanen H., Truijens M. (2017). Using tri-axial accelerometry in daily elite swim training practice. Sensors.

[11] Benson L.C., Clermont C.A., Bošnjak E., Ferber R. (2018). The use of wearable devices for walking and running gait analysis outside of the lab: A systematic review. Gait Posture.

[12] Camomilla V., Bergamini E., Fantozzi S., Vannozzi G. (2018). Trends supporting the in-field use of wearable inertial sensors for sport performance evaluation: A systematic review. Sensors.

[13] Chesher S.M., Rosalie S.M., Chapman D.W., Charlton P.C., van Rens F.E.C.A., Netto K.J. (2024). A single trunk-mounted wearable sensor to measure motor performance in triathletes during competition. Proc. Inst. Mech. Eng. P J. Sport Eng. Technol..

[14] Delhaye E., Bouvet A., Nicolas G., Vilas-Boas J.P., Bideau B., Bideau N. (2022). Automatic swimming activity recognition and lap time assessment based on a single IMU: A deep learning approach. Sensors.

[15] Jowitt H.K., Durussel J., Brandon R., King M. (2020). Auto detecting deliveries in elite cricket fast bowlers using microsensors and machine learning. J. Sports Sci..

[16] Murray N.B., Black G.M., Whiteley R.J., Gahan P., Cole M.H., Utting A., Gabbett T.J. (2017). Automatic detection of pitching and throwing events in baseball with inertial measurement sensors. Int. J. Sports Physiol. Perform..

[17] Hendry D., Chai K., Campbell A., Hopper L., O’Sullivan P., Straker L. (2020). Development of a human activity recognition system for ballet tasks. Sports Med..

[18] Hulin B.T., Gabbett T.J., Johnston R.D., Jenkins D.G. (2017). Wearable microtechnology can accurately identify collision events during professional rugby league match-play. J. Sci. Med. Sport.

[19] McKay A.K.A., Stellingwerff T., Smith E.S., Martin D.T., Mujika I., Goosey-Tolfrey V.L., Sheppard J., Burke L.M. (2022). Defining training and performance calbre: A participant classification framework. Int. J. Sports Physiol. Perform..

[20] Farrahi V., Rostami M. (2024). Machine learning in physical activity, sedentary, and sleep behaviour research. J. Act. Sedentary Sleep Behav..

[21] Wang B. (2024). Signal processing based on Butterworth filter: Properties, design, and applications. High. Sci. Eng. Technol..

[22] Ramos-Aguilar R., Olvera-López J.A., Olmos-Pineda I., Snchez-Urrieta S., Martín-Ortiz M. (2019). Parameter experimentation for epileptic seizure detection in EEG signals using Short-Time Fourier transform. Res. Comput. Sci..

[23] Mateo C., Talavera J.A. (2018). Short-Time Fourier transform with the window size fixed in the frequency domain. Dig. Signal Process..

[24] Eertink J.J., Heymans M.W., Zwezerijnen G.J.C., Zijlstra J.M., de Vet H.C.W., Boellaard R. (2022). External validation: A simulation study to compare cross-validation versus holdout or external testing to assess the performance of clinical prediction models using PET data from DLBCL patients. EJNMMI Res..

[25] Rodríguez L., Veiga S., García I., González-Ravé J.M. (2021). Stroking rates of open water swimmers during the 2019 FINA World Swimming Championships. Int. J. Environ. Res. Public Health.

[26] Landers G.J., Blanksby B.A., Rackland T. (2011). Cadence, stride rate and stride length during triathlon competition. Int. J. Exerc. Sci..

[27] Wundersitz D.W.T., Gastin P., Richter C., Robertson S.J., Netto K.J. (2014). Validity of a trunk-mounted accelerometer to assess peak accelerations during walking, jogging and running. Eur. J. Sport Sci..

[28] Banos O., Galvez J., Damas M., Pomares H., Rojas I. (2014). Window size impact in human activity recognition. Sensors.

[29] Bianchi V., Ambrosini L., Presta V., Gobbi G., de Munari I. (2022). Prediction of kick count in triathletes during freestyle swimming session using inertial sensor technology. Appl. Sci..

[30] Gentile A.M., Carr J.H., Shepherd R.D. (2000). Skill acquisition: Action, movement, and neuromotor processes. Movement Science: Foundations for Physical Therapy.

[31] Bouillod A., Grappe F. (2018). Physiological and biomechanical responses beween seated and standing positions during distance-based uphill time trials in elite cyclists. J. Sports Sci..

[32] Zignoli A., Biral F. (2020). Prediction of pacing and cornering strategies during cycling individual time trials with optimal control. Sports Eng..

